# Supplementary material for: Talin rod domain–containing protein 1 (TLNRD1) is a novel actin-bundling protein which promotes filopodia formation
Source: J Cell Biol. 2021 Jul 15;220(9):e202005214. doi: 10.1083/jcb.202005214 (PMC8287531; doi:10.1083/jcb.202005214)
Supplement: Table S1 — shows data collection and refinement statistics for TLNRD1-FL and TLNRD1-4H domains. [file JCB_202005214_TableS1.docx]

Supplementary Table 1. Data collection and refinement statistics for TLNRD1 full-length and 4-helix domain

| **Data collection** | **TLNRD1-FL** | **TLNRD1-4H** |
| --- | --- | --- |
| Synchrotron and Beamline | Soleil Proxima-1 | Soleil Proxima-1 |
| Space group | *P*2_1_ | *I*4_1_22 |
| Molecule/a.s.u  Cell dimensions | 2 | 1 |
| *a*, *b*, *c* (Å) | 69.17, 58.04, 84.32 | 114.59, 114.59, 59.40 |
| α, β, γ (°) | 90, 106.10, 90 | 90, 90, 90 |
| Resolution (Å) | 60.22 – 2.30  (2.38 – 2.30)* | 57.30 – 2.19  (2.31 – 2.19) |
| *R*_merge_ | 0.087 (0.682) | 0.130 (1.034) |
| *I* / σ*I* | 5.7 (1.3) | 12.5 (2.6) |
| *CC(1/2)* | 0.994 (0.831) | 0.996 (0.938) |
| Completeness (%) | 98.4 (98.7) | 100 (99.9) |
| Redundancy | 2.8 (2.8) | 13.4 (13.6) |
|  |  |  |
| **Refinement** |  |  |
| Resolution (Å) | 2.30 | 2.19 |
| No. reflections | 27997 (2669) | 10464 (2428) |
| *R*_work_ / *R*_free_ | 0.26/0.31 | 0.22/0.26 |
| No. atoms |  |  |
| Protein | 9047 | 1903 |
| Water | 70 | 38 |
| *B*-factors (Å^2^) |  |  |
| Protein | 74.25 | 72.05 |
| Water | 50.47 | 56.53 |
| R.m.s. deviations |  |  |
| Bond lengths (Å) | 0.004 | 0.003 |
| Bond angles (°) | 0.792 | 0.620 |
| Ramachandran plot |  |  |
| Favoured/allowed/  outlier (%) | 95.20/3.64/1.16 | 96.69/1.65/1.65 |
| Rotamer |  |  |
| Favoured/poor (%) | 88.31/4.18 | 96.12/0.97 |
| MolProbity scores |  |  |
| Protein geometry | 1.75 (97^th^) | 1.01 (100^th^) |
| Clash score all atoms | 1.88 (100^th^) | 1.05 (100^th^) |
| PDB accession no. | 6XZ4 | 6XZ3 |

*Values in parentheses are for highest-resolution shell.
